# Case report: severe myoclonus associated with oral midodrine treatment for hypotension

**DOI:** 10.1097/MD.0000000000021533

**Published:** 2020-10-02

**Authors:** Xiaolan Ye, Bai Ling, Jian Wu, Shujuan Wu, Yan Ren, Hongjuan Zhang, Feifeng Song, Zixue Xuan, Maosheng Chen

**Affiliations:** a Department of Pharmacy, Zhejiang Provincial People's Hospital; b Department of Pharmacy, People's Hospital of Hangzhou Medical College, Hangzhou 310014; c Department of Pharmacy, The First People's Hospital of Yancheng City, Yancheng 224005, Jiangsu; d State Key Laboratory for the Diagnosis and Treatment of Infectious Diseases, National Clinical Research Center for Infectious Diseases, Collaborative Innovation Center for Diagnosis and Treatment of Infectious Diseases, First Affiliated Hospital, College of Medicine, Zhejiang University, 79 Qingchun Rd.; e Department of Pharmacy, Third Affiliated hospital of Wenzhou Medical University; f Department of Nephrology, Zhejiang Provincial People's Hospital; g Department of Nephrology, People's Hospital of Hangzhou Medical College, Hangzhou 310014, People's Republic of China.

**Keywords:** midodrine, myoclonus, hypotension, chronic kidney disease

## Abstract

**Rationale:**

Midodrine is widely used in the treatment of hypotensive states, there have been no reports of myoclonus associated with midodrine use in hypotension with chronic kidney disease.

**Patient concerns:**

We report a 58-year-old female patient with chronic kidney disease (CKD) presenting with involuntary tremor 2 h after taking midodrine, which became more frequent after 6 h. Brain CT and neurological examination did not yield findings of note. Blood chemistry showed serum albumin of 3.1 g/L, ALT of 19 U/L, AST of 22 U/L, SCr of 273.9 μmol/L, K^+^ of 2.94 mmol/L, Ca^2+^ of 1.63 mmol/L, and Mg^2+^ of 0.46 mmol/L. Her BP was maintained at 83–110/56–75 mmHg. Her urine volume was 600–1000 mL/d, and her heart rate was within a range of 90–100 beats/min.

**Diagnosis:**

Chronic kidney disease (CKD), hypotension, metabolic acidosis, hypocalcemia, hypokalemia, and hypomagnesemia.

**Interventions:**

Midodrine treatment was stopped and the patient was treated with intravascular rehydration and furosemide. Myoclonus ceased one day after midodrine withdrawal.

**Lessons:**

Oral midodrine is widely used in the treatment of orthostatic hypotension, recurrent reflex syncope and dialysis-associated hypotension and the adverse effects are mostly mild. However, clinicians should be alert for midodrine-induced myoclonus, especially in patients with CKD.

## 1 Introduction

Midodrine is widely used in the treatment of hypotensive states, particularly in orthostatic hypotension.[^1^,^2^] To our knowledge, this is the first reported case of myoclonus caused by midodrine. The pharmacological mechanisms leading to this adverse drug reaction (ADR) have not been established.

## 2 Case Report

A 58-year-old female patient with chronic kidney disease (CKD) was treated with laser lithotripsy. After 20 days, her serum creatinine (SCr) had increased from 200 to 280.2 μmol/L, at which point she was admitted to the hospital. SCr continued to increase to 393.0 μmol/L over the course of the next 16 days. Upon admission, she was fatigued, weak, and consistently dizzy. Blood pressure (BP) was 106/63 mmHg. Blood chemistry showed albumin of 3.1 g/L, ALT at 19 U/L, AST at 22 U/L, SCr at 273.9 μmol/L, K^+^ at 2.94 mmol/L, Ca^2+^ at 1.63 mmol/L, and Mg^2+^ at 0.46 mmol/L. She was diagnosed with metabolic acidosis, hypocalcemia, hypokalemia, and hypomagnesemia, and was treated with intravenous calcium gluconate, oral potassium sodium hydrogen citrate granules, potassium, and magnesium aspartate. Three days after treatment, the patient still complained of dizziness and physical weakness. Her BP was maintained at 83–110/56–75 mmHg, her urine volume was 600–1000 mL/d, and her heart rate had a range of 90–100 beats/min. Since she suffered from hypotension, she was given oral midodrine (2.5 mg three times daily). She took midodrine for the first time at 19:00 pm, and felt intermittent involuntary tremors after 2 h. Tremors became more frequent after 6 h. There were no observations of salivation, urination, or biting of the tongue, and she was able to obey verbal commands. The patient reported that she could not control her arms to keep them still. Blood gas analysis indicated a pH of 7.46, with Ca^2+^ of 0.93 mmol/L. Despite continuous supplementary intravenous calcium, the symptoms were not relieved. After taking midodrine for the second and third time, the patient had a generalized myoclonic seizure and paresthesia, described as the sensation of crawling ants on her skin. She reported that the clonus was less painful if she lay down, and she was unable to walk by herself. There were no additional significant abnormalities in her brain CT scan or neurological examination, although she was dehydrated from hemodialysis. She was given oral diazepam to relieve anxiety, but this was also ineffective. After evaluating the patient's medicines and manifestations, a clinical pharmacist suggested that the myoclonus reflected a rare ADR of midodrine, and midodrine was withdrawn immediately. Since the patient's blood pressure was still low, intravascular rehydration and furosemide were given without an alpha-1-receptor antagonist. Her symptoms improved gradually, and myoclonus ceased a day after midodrine withdrawal, although her ionized calcium remained abnormal. Myoclonus did not recur during hospitalization, and the patient did not report any further symptoms at the outpatient clinic one month later. The event was evaluated as Naranjo Scale^[7]^ is 7, and we therefore suspect it to be an adverse drug reaction.

## 3 Discussion

Midodrine is a direct-acting sympathomimetic with selective alpha-1-agonist activity. Its main active moiety is its major metabolite, deglymidodrine, which reaches peak plasma concentration about 1 h after oral administration, the half-life (t_1/2_) of deglymidodrine is about 3 h, and the duration of drug action is 4–6 h.[^2^,^3^] Midodrine is mainly excreted through the urine as metabolites and a small amount of unchanged form.[^2^,^3^,^4^]

Midodrine is widely used in the treatment of orthostatic hypotension, recurrent reflex syncope, and dialysis-associated hypotension,[^1^,^2^,^3^,^4^,^5^] and raises blood pressure by acting on arterial and venous smooth muscle contraction.^[6]^ Notable side effects include supine and sitting hypertension, paresthesia, pruritus (mainly of the scalp), goosebumps, chills, urinary urge, urinary retention, and urinary frequency. Though the FDA label of midodrine mentions leg cramps as a rare ADR,^[4]^ we were unable to find other cases reports of generalized myoclonus in Medline, PubMed, or Clinical Trial Data Review. Despite this rareness, the Naranjo Scale^[7]^ indicated score of 7, or a probable relationship between the patient's development of myoclonus and use of midodrine (Table 1).

**Table 1:**
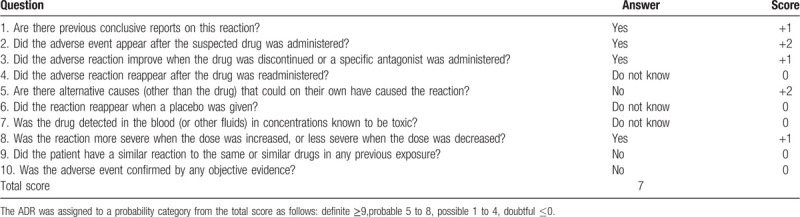
The Naranjo adverse drug reaction probability scale.

The exact mechanisms this ADR cannot yet be characterized. Several studies have indicated that anxiety, fear, restlessness, insomnia, confusion, irritability, psychotic states, and other central nervous system effects may occur with all sympathomimetics. Some sympathomimetics which do not cross the blood-brain barrier appear to be a somatic response to their central effects. In addition, epinephrine can facilitate neuromuscular transmission followed by a prolonged rapid stimulation of motor nerves.^[2]^ Some studies have found that the stimulation of receptors causes a more rapid increase in transmitter release from the somatic motor neuron, perhaps due to the enhanced influx of Ca^2+^. These responses are most likely mediated by alpha-1 receptors.^[2]^

In treating or preventing midodrine alpha-mediated ADRs such as hypertension, a rapidly-acting alpha blocker such as phentolamine may be given. However, it is not known whether this could affect ADRs such as myoclonus, especially for patients with hypotension. As such, supportive care may be more suitable. In our case, the patient had chronic renal insufficiency, with decreased urine output at admission, and midodrine excretion was delayed. As a result, the myoclonus ADR lasted for one day after drug withdrawal.

## 4 Conclusion

This case is the first reported case of myoclonus as a serious and unexpected adverse drug reaction to midodrine. Our experience suggests that careful attention should be given to patients taking midodrine, especially for those with CKD, since they may be more susceptible to midodrine side effects.

## Author contributions

**Conceptualization:** Xiaolan Ye, Bai Ling, Yan Ren, Hongjuan Zhang.

**Data curation:** Hongjuan Zhang, Feifeng Song, Zixue Xuan.

**Investigation:** Xiaolan Ye, Shujuan Wu, Feifeng Song, Zixue Xuan, Maosheng Chen.

**Methodology:** Xiaolan Ye, Yan Ren, Maosheng Chen.

**Project administration:** Xiaolan Ye.

**Resources:** Xiaolan Ye, Maosheng Chen.

**Supervision:** Hongjuan Zhang.

**Writing – original draft:** Xiaolan Ye.

**Writing – review & editing:** Bai Ling, Jian Wu, Maosheng Chen.
